# Convolutional Neural Networks to Assess Steno-Occlusive Disease Using Cerebrovascular Reactivity

**DOI:** 10.3390/healthcare11162231

**Published:** 2023-08-08

**Authors:** Yashesh Dasari, James Duffin, Ece Su Sayin, Harrison T. Levine, Julien Poublanc, Andrea E. Para, David J. Mikulis, Joseph A. Fisher, Olivia Sobczyk, Mir Behrad Khamesee

**Affiliations:** 1 Department of Mechanical and Mechatronics Engineering, University of Waterloo, Waterloo, ON N2L 3G1, Canada; ydasari@uwaterloo.ca; 2 Department of Physiology, University of Toronto, Toronto, ON M5S 1A8, Canada; 3 Department of Anesthesia and Pain Management, University Health Network, Toronto, ON M5G 2C4, Canada; 4 Joint Department of Medical Imaging and the Functional Neuroimaging Laboratory, University Health Network, Toronto, ON M5G 2C4, Canada; 5 Institute of Medical Sciences, University of Toronto, Toronto, ON M5S 1A8, Canada

**Keywords:** blood oxygenation level-dependent magnetic resonance imaging (BOLD-MRI), cerebrovascular reactivity (CVR), convolutional neural networks (CNNs), deep learning, medical image analysis, steno-occlusive disease (SOD)

## Abstract

Cerebrovascular Reactivity (CVR) is a provocative test used with Blood oxygenation level-dependent (BOLD) Magnetic Resonance Imaging (MRI) studies, where a vasoactive stimulus is applied and the corresponding changes in the cerebral blood flow (CBF) are measured. The most common clinical application is the assessment of cerebral perfusion insufficiency in patients with steno-occlusive disease (SOD). Globally, millions of people suffer from cerebrovascular diseases, and SOD is the most common cause of ischemic stroke. Therefore, CVR analyses can play a vital role in early diagnosis and guiding clinical treatment. This study develops a convolutional neural network (CNN)-based clinical decision support system to facilitate the screening of SOD patients by discriminating between healthy and unhealthy CVR maps. The networks were trained on a confidential CVR dataset with two classes: 68 healthy control subjects, and 163 SOD patients. This original dataset was distributed in a ratio of 80%-10%-10% for training, validation, and testing, respectively, and image augmentations were applied to the training and validation sets. Additionally, some popular pre-trained networks were imported and customized for the objective classification task to conduct transfer learning experiments. Results indicate that a customized CNN with a double-stacked convolution layer architecture produces the best results, consistent with expert clinical readings.

## 1. Introduction

Cerebrovascular diseases include conditions and disorders that affect the blood vessels and cerebral blood flow (CBF) [1]. These diseases affect a significant population worldwide, with stroke being the leading cause of long-term disability and the second-leading cause of death. Globally, over 12.2 million new stroke cases are reported annually, with 7.6 million of these categorized as ischemic stroke. Additionally, the same statistical report predicts that 1 in 4 people over the age of 25 will have a stroke in their lifetime [2]. Steno-occlusive disease (SOD) involves regional arterial occlusion (blockage) or stenosis (narrowing) in the brain and is the most common cause of stroke, with arterial blockage accounting for about 85% of stroke cases [3,4]. Research also suggests that SOD patients are at a high risk of developing recurrent ischemic stroke [5].

Blood Oxygenation Level Dependent (BOLD) imaging maps the differences in CBF when a vasodilatory stimulus is administered and assumes that metabolic activity in the brain is stable during data acquisition [6]. Cerebrovascular Reactivity (CVR) is a provocative test that measures the ability of smooth muscle in the arterial walls to adjust the diameter of the vessels, independent of the effects of vessel diameter adjustments influenced by blood pressure changes or changes in neural and glial activity [7]. This test is analogous to a cardiac stress test where the patient exercises and the change in coronary blood flow are observed.

CVR closely relates to the brain vasculature’s health and can highlight cerebrovascular diseases such as Alzheimer’s disease (AD), SOD, stroke, and traumatic brain injury [8]. In patients with SOD, the administration of an external stimulus leads to CBF redistribution and can result in regional steal physiology [9]. Therefore, analyzing information about the severity and spatial location of abnormal CVR at the tissue level can play a vital role in the early diagnosis and management of SOD.

Currently, CVR maps are analyzed by a team of experts who visually examine CVR distribution across brain regions and evaluate the brain vasculature conditions. Motivated by the extensive prevalence of cerebrovascular diseases and the well-established success of deep learning in medical imaging, a convolutional neural network (CNN)-based clinical decision support system to facilitate CVR assessment would be welcomed. The automatic feature extraction adds several benefits to the CVR analysis workflow. The proposed model accounts for the skull-based susceptibility artifact, which are otherwise manually removed. It is also agnostic to the orientation of the maps in the mosaic stack since the training set has samples that were flipped horizontally.

To optimize the assessment of the effects of SOD on cerebral blood flow regulation in individual patients, a method enabling precisely reproducible carbon dioxide (CO_2_) stimuli was applied using prospective end-tidal targeting of CO_2_ during high temporal resolution whole brain BOLD-MRI. The CVR maps thus generated were used for training and testing the CNNs. The proposed model can be used by participating institutions as a research tool for classifying CVR maps generated using the clinical workflow used in this work and for advancing the application of deep learning and CNNs in CVR research.

### 1.1. CVR: Clinical Workflow

Clinicians have administered different types of stimuli for CVR studies, including, transient reduction in mean arterial blood pressure, chemical injections, and changes in arterial partial pressure of CO_2_ [10]. The CVR maps used in this work were obtained by administering a non-invasive stimulus, involving manipulation of the end-tidal partial pressures of CO_2_ and O_2_ gas concentrations in spontaneous breath studies (prospective end-tidal targeting) as the vasoactive stimulus and mapping the corresponding changes [11]. The gas concentration manipulation was accomplished using a computer-controlled gas blender called the RespirAct RA-MR [12], and a step-and-ramp stimulus protocol was administered [13]. 

CVR is measured as the ratio of the change in BOLD signal(s) (ΔBOLD) with respect to the change in end-tidal partial pressure of CO_2_ (ΔP_ET_CO_2_) [14], as shown in the equation below:

(1) $$CVR=\frac{\Delta BOLD}{\Delta P_{ET}{CO}_{2}}.$$

**Figure 1:**
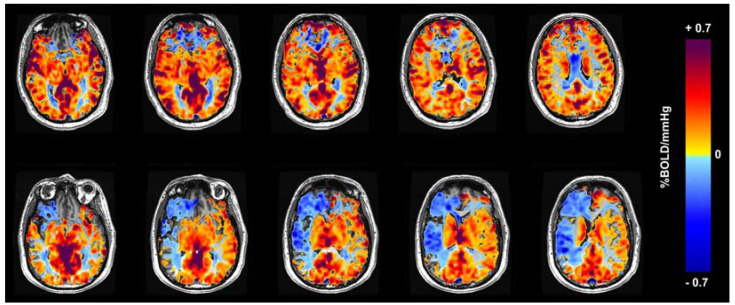
Colored CVR maps overlaid on the corresponding anatomic T1 weighted images (note: MRI images are displayed from the perspective of the feet looking up, so the right side of the brain is on the left side of the image). Positive CVR is yellow−red and negative CVR (steal physiology) is blue. Top row: normal healthy control. There is a susceptibility artifact from the skull base, which causes artifactually decreased CVR in the anterior inferior frontal lobes. Bottom row: patient with right−sided steno−occlusive disease and extensive steal physiology in the right middle cerebral artery (MCA) territory (blue which is on the left side of the images).

The voxel-by-voxel CVR values calculated across the brain regions are co-registered on the corresponding anatomical scans. For the CVR maps used in this work, an increase in CVR is indicated by positive values and is colored in shades of yellow, orange, and red. Similarly, reduced CVR is indicated by negative values and is colored in shades of blue. Figure 1 shows two sample CVR maps obtained from a healthy control subject and a patient with SOD, highlighting the normal and abnormal CVR distribution using the specified color scale.

### 1.2. Convolutional Neural Networks

Deep Learning, a subset of Artificial Intelligence (AI), is a type of representation learning that automatically discovers important representations (called features) needed to make decisions from raw input data as a part of its learning algorithm, eliminating the need to manually pre-process the raw data [15]. At its core, deep learning models are made up of artificial neural networks that use a network of functions to establish interpretations from features and map them to a specific output [16].

Convolutional Neural Networks (CNNs) are specialized deep learning models that use a linear mathematical operator called convolution, in their convolution layers [17]. CNNs are the most popular deep learning algorithm used in visual learning tasks because it significantly reduces the number of training parameters (dimensionality reduction) while preserving local image relations. The convolution layers extract meaningful representations from the raw input data, which are then fed into the fully connected layers for the objective task. Therefore, a CNN can be described as a combination of feature extractor-classifier models. 

A convolution layer performs a convolution operation on the input image using a pre-defined filter. Other layers in a CNN can be a pooling layer, a dropout layer, a normalization layer, a loss layer, and so on [18]. A convolution operation is generally denoted with an asterisk. For input, *I*(*t*) with an independent variable *t*, a convolution operation with a filter *K*(*a*), to obtain a feature map *s*(*t*) is shown in the equation below [15]:

(2) $$s\left(t\right)=(I*K)(t).$$

An activation function (or transfer function) is used to adjust or map the generated output from a layer on a new scale [19]. Two types of non-linear activation functions are used in this work, the Sigmoid and the Rectified Linear Unit (ReLU) activation functions [20]. A pooling layer is typically used after the convolution layers to down-sample the feature maps, reducing their complexity. In image-related tasks, it reduces the width and height of the feature maps. Once the feature maps have extracted important representations from the input, the fully connected layer(s) compute the probability score for the objective classification [21].

Transfer learning is a widely used concept in deep learning where parametric-level knowledge is transferred between networks. The objective of such experiments is to use a pre-trained network, originally trained on partially related or unrelated datasets, and use these weights to accomplish the target classification task [22].

### 1.3. Article Structure

The article is organized as follows. Section 2 discusses the recent work and advancements related to the application of deep learning in medical imaging and CVR studies, focusing on CNNs. Section 3 explains data preparation and pre-processing, network design methodology, and the deep learning concepts implemented. Section 4 presents the quantitative results obtained from the experiments and compares the networks’ performance. Section 5 discusses the key findings of this research, the proposed network, and outlines the future scope. Finally, Section 6 draws conclusions from this work.

## 2. Relevant Work

Deep Learning has been successfully implemented in wide-ranging medical imaging tasks, for example, image segmentation and classification, and computer-aided disease diagnosis [23,24]. In radiology, the spatial structures of the organ are pivotal in classifying healthy versus unhealthy cases. Therefore, CNNs are particularly effective in this domain because they preserve local spatial relationships when filtering input images [25].

Farooq et al. developed a deep CNN-based pipeline for a four-way classification of brain MRI scans. The experiments were conducted using the Alzheimer’s Disease Neuroimaging Initiative (ADNI) dataset and obtained state-of-the-art results to achieve a prediction accuracy of 98.8%. The dataset was categorized into a healthy control group, Alzheimer’s Disease (AD), Mild Cognitive Impairment (MCI), and Late Mild Cognitive Impairment (LMCI) [26]. 

Chen et al. implemented CNNs to predict the cerebrovascular reserve in Moyamoya disease in a simultaneous [15O]-water positron emission tomography (PET)/MRI before acetazolamide administration on a dataset consisting of 24 patients and 12 healthy control subjects. Two models were used, one with both PET and MRI and another with only MRI. Both models successfully identified the regions of impaired cerebrovascular reserve, both achieving an area of 0.95 under the receiver operating characteristic curve [27]. 

Hussein et al. used datasets from cerebral blood flow (CBF)-based studies and proposed a multi-task learning workflow consisting of MRI-to-PET translation, followed by disease diagnosis. The work reported an average classification accuracy of 96.38% for three disorders including Moyamoya disease, SOD, and stroke [28].

Hashemzehi et al. proposed a hybrid model using a neural autoregressive distribution estimation (NADE) and a CNN for brain tumor detection. The model was tested with 3064 T1-weighted contrast-enhanced images and achieved a 95% accuracy in classifying three types of tumors [29]. 

Mejis et al. presented a CNN model to detect image-level intracranial anterior circulation artery occlusions using 4D-CTA imaging. The model was trained using 214 samples and obtained an accuracy of 92% on the test set of 279 samples [30].

Hou et al. used deep learning for resting-state vascular imaging to detect vascular abnormalities using cerebrovascular reactivity (CVR) and bolus arrival time (BAT) maps of the brain that were obtained using resting-state fMRI studies [31].

In addition to the detection, classification, and prediction of the cerebrovascular diseases discussed above, Zhu et al. provide a comprehensive review of deep learning-based generation and enhancement of stroke imaging, using Computed Tomography (CT) and MRI [32].

Transfer learning has also proven very effective with medical datasets. Talo et al. successfully fine-tuned ResNet34 for the binary classification of 613 MR images, using techniques such as image augmentation and optimal learning rate finder, and achieved a five-fold classification accuracy of 100% [33]. Maqsood et al. used AlexNet for the classification of 3D MRI datasets, including both segmented and unsegmented images, and achieved an accuracy of 92.85% for the multi-class classification of unsegmented images [34]. Yuan et al. proposed a novel transfer learning-based multiparametric MR model for prostate cancer classification, achieving an accuracy of 86.92% [35].

Mohsen et al. applied transfer learning for domain adaptation for MRI scanner agnostic studies. This research investigated white matter hyperintensity segmentation using CNNs and concluded that transferring pre-trained network weights of one set of MRI images to a different MRI dataset outperformed a new network trained from scratch [36].

Many conventional machine learning models have also been successful in CVR-related learning tasks. Spencer et al. used Support Vector Machines (SVMs) to identify cerebrovascular impairment using CVR-weighted hypercapnic BOLD and other MRI techniques, achieving high performance (specificity = 0.67; sensitivity = 0.75) [37]. Kloppel et al. also used SVMs to detect AD using structural MRI and reported an accuracy of 96% [38]. Evangelia et al. investigated Linear Discriminant Analysis (LDA) with Fisher’s discriminant rule, k-Nearest Neighbor (k-NN), and nonlinear SVMs to classify brain tumors using MRI datasets. The binary SVM classification for discrimination of metastases from gliomas achieved accuracy, sensitivity, and specificity of 85%, 87%, and 79%, respectively [39]. Bahadure et al. used SVMs for the binary classification of normal versus abnormal tissues using Berkeley wavelet transformation (BWT) based brain tumor segmentation and achieved 96.51% accuracy, 94.2% specificity, and 97.72% sensitivity [40].

## 3. Materials and Methods

### 3.1. Data Analysis and Pre-Processing

**Figure 2:**
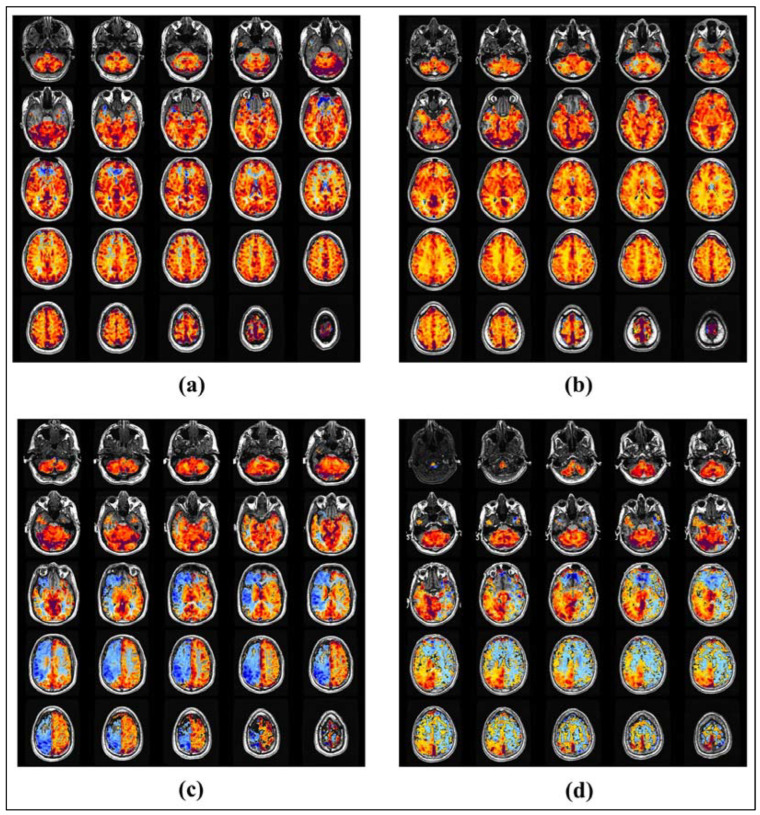
5 × 5 mosaic images of the CVR maps from the input dataset. CVR maps in samples (**a**), and (**b**) were measured in healthy subjects and the maps in samples (**c**), and (**d**) were measured in SOD patients. As shown, there is a reduced CVR response in the right and left hemispheres of the brain in samples (**c**), and (**d**), respectively.

The input dataset used for training the networks contained 2-dimensional RGB images of the CVR maps in axial view, stacked in a 5 × 5 mosaic format. Each input image displayed CVR maps across the brain with a 5-slice spacing, plotted in the standard coordinate space, and had a resolution of 1280 × 1280 × 3 pixels [41]. Using the CVR maps in a mosaic format allowed the networks to be trained with comprehensive and sufficient details to make judgments on the overall brain vasculature’s health. Training the networks using individual slices of CVR maps may lead to the network learning localized attributes, native to individual slices. The input dataset was obtained from a diverse patient cohort from studies conducted at our sponsoring institute and was labeled by a team of experts. Figure 2 shows four sample input images, two belonging to healthy control subjects (a, b), and two to SOD patients (c, d).

**Figure 3:**
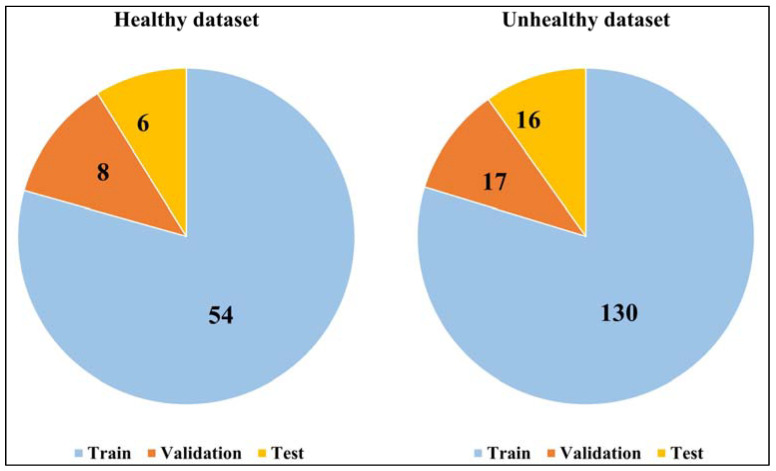
Distribution of the original dataset (68 healthy, and 163 unhealthy samples) into the train, validation, and test subsets. The splitting ratio within each category (healthy and unhealthy) is roughly 80%-10%-10%, as shown.

The CVR dataset was obtained by administering a step-and-ramp CO_2_ stimulus protocol using prospective end-tidal targeting [13]. The original dataset contains 68 healthy control subjects and 163 patients, which was divided into three sub-categories to train, validate, and test the networks, split in a ratio of 80%-10%-10%, respectively, as shown in Figure 3.

Deep learning models heavily rely on the quantity of data available for training the networks [42]. The limited training samples available for this study can be considered a small dataset. Therefore, to address the data scarcity, artificial features were added to the training dataset using data augmentation techniques. Additionally, to accommodate some of the real-world differences in CVR maps that arise due to experimental settings, small augmentations were applied to the validation set as well. However, to maintain a standardized CVR assessment protocol, the augmentations in both sets were small-scaled and did not affect the original attributes of the mosaic images. The augmentation techniques used included rotation (0–20%), shearing (0–20%), brightness modifications (30–100%), and horizontal flips. The test set was unaltered. The final number of samples in each dataset after data augmentation is shown in Table 1.

### 3.2. CNN Design

The optimization of CNNs mainly involves fine-tuning the network’s hyperparameters (configurable values set before the training begins) during successive trials of training. The network automatically updates its parameters (weights and biases) during the learning process [43].

The CNN design and optimization were conducted using Python (v. 3.10.4), Tensorflow (v. 2.9.1) [44], and Keras (v. 2.9.0) [45] libraries. The computational setup consisted of an Intel XII Gold 5218 dual-core 2.3 GHz processor, with 256 GB RAM, and a 64-bit Windows 10 operating system, and the experiments were run as CPU processes. The design strategy involved training a small, shallow CNN, and using its performance as a benchmark to improve the network configurations and settings. The network hyperparameters were further fine-tuned to develop more optimized networks. The trained model details such as the network architecture, weights, and state of the trained models were saved for testing and future use.

All the customized networks investigated were trained using the Adam optimization algorithm [46]. ReLU activation function [47] was used with the convolution layers, and the max pooling operation [48] was used after the convolution layer(s), with a pool size of 2 × 2. After the feature extraction process, a flatten layer was used to flatten the multi-dimensional input tensor into a single dimension that could be fed into a fully connected layer. The hidden fully connected layers used the ReLU activation function, and the output fully connected layer used a sigmoid activation function. The binary cross-entropy loss function was used, and the accuracy was monitored after each epoch. The networks were trained for up to 200 epochs, with the early stopping callback. For early stopping, the validation loss was monitored with a patience of 10 epochs [49]. 

With limited training samples, the relationships that a neural network learns can be the result of sampling noise, which does not exist in real test data. This leads to overfitting [50]. To address this, dropout layers were added to the network architecture. Dropout [51] is a widely used regularization technique where units along with their connections are dropped from the network. This happens only during training. Another effective normalization technique that reparametrizes deep networks called batch normalization (also called batch norm) was investigated. It applies transformations to normalize the inputs fed to the subsequent layer [52].

The different hyperparameters experimented with and fine-tuned during the network optimization are listed in Table 2.

### 3.3. Transfer Learning

Some of the popular pre-trained networks, trained on the ImageNet dataset (ImageNet Large Scale Visual Recognition Challenge; ILSVRC) [53], were used to implement transfer learning using the CVR dataset. The networks investigated were EfficientNetB0 [54], InceptionV3 [55], ResNet50 [56], and VGG [57].

These networks were imported with their pre-trained weights using Keras libraries, and then modified to suit the objective binary classification task by freezing all the layers and replacing the classifiers. This workflow allowed leveraging the features learned by the pre-trained models and making predictions on the CVR dataset [58].

The original images were downsized to 224 × 224 pixels, and a batch size of 32 was used while using the early stopping callback that monitored the validation loss with a patience of 10 epochs. 

For InceptionV3, the base model was instantiated with the pre-trained weights and all the layers were frozen, and the ImageNet classifier was excluded. A flatten layer was added after the last fully connected layer, making the output shape (None, 51200). Following this, another fully connected layer was added with 1024 neurons and a ReLU activation function, followed by a dropout layer (dropout rate = 50%), and an output layer with a sigmoid activation function.

Similarly, for EfficientNetB0, ResNet50 and VGG16, the pre-trained networks were imported while excluding the classifier. Only one fully connected output layer was added on top of the frozen layers with a sigmoid activation function.

## 4. Results 

As discussed in Section 3, the customized CNNs were trained using the mosaic CVR dataset for up to 200 epochs, with a learning rate of 0.001, the Adam optimizer, and an early stopping callback. While investigating the effects of batch size, it was observed that increasing the batch size improved the model performance and reduced the noise in the validation loss. Based on the results and recommendations from Bengio [59], a batch size of 32 was used.

### 4.1. Baseline Experiments

The baseline model consisted of an input layer, a convolution layer with 8 filters, a fully connected layer with 8 neurons, and an output layer with 1 neuron. The first series of experiments investigated the effect of input image resolution, comparing downsized resolutions of 256 × 256 and 512 × 512 pixels. The results indicated that an input resolution of 256 × 256 pixels produced optimal results.

However, the overall performance of this network was poor, and it achieved training and validation accuracy of 52.3% and 52.5%, respectively. 

### 4.2. Dropout Regularization and Batch Normalization

To address the overfitting, regularization techniques were introduced in the network architecture. When dropout was proposed, it was only used with fully connected layers and not convolution layers [51]. However, more recent studies have demonstrated that this could indeed improve performance [60].

Two dropout rates were investigated with the baseline model: 0.2 (dropping 20% nodes), and 0.5. A dropout layer was also added after the convolution layer to test the contradicting rules about this practice. Experiments demonstrated that using dropout layers with a dropout rate of 0.5 after both the convolution layer and the hidden fully connected layer produced the best results. 

Finally, adding batch normalization layers after the convolution layer and the hidden fully connected layer further improved the results. The configuration of the dropout layer, batch norm layer, and activation function is again a topic of debate [61]. This study presents a configuration best suited for the dataset in consideration.

The best-performing network had the following architecture: input layer, convolution layer (8 filters), batch norm layer, dropout layer (rate = 0.5), fully connected layer (8 nodes), batch norm layer, dropout layer (rate = 0.5), and output layer.

### 4.3. Network Width and Depth

Using the inferences from the baseline experiments, the network width and depth were investigated next. In this series of sensitivity analyses, the widths of existing convolution and hidden fully connected layers were increased from 8 to 64. For network depth experiments, additional convolution layers and fully connected layers, along with dropout and batch norm layers were added.

### 4.4. Advanced Hyperparameters

Some of the advanced hyperparameters were experimented with using the best-performing networks. The results indicated that the kernel size had an insignificant effect on the overall network performance. Therefore, a 3 × 3 kernel size is recommended for this CVR dataset.

Three learning rates were investigated: 0.001 (default), 0.0005, and the ReduceLROnPlateau [62] callback. ReduceLROnPlateau reduced the learning rate when the selected performance metric stops to improve. Based on the results, the default learning rate of 0.001 is recommended for the CVR dataset used in this work.

### 4.5. Network Performance Comparison

A comparison of the accuracy and loss values obtained from experimenting with the hyperparameter settings is shown in Table 3. The table describes the network architecture, the total number of parameters, the number of epochs the models trained for, and the performance metrics.

The generalization capabilities of a network can be evaluated using its performance on unseen data in the test set. In a binary classification task, this is carried out using the metrics discussed below.

Based on the prediction made by the classification model on an input, the outcomes can be classified into four groups:

1. True Positive (TP): Number of samples classified correctly as yes or success.
2. True Negative (TN): Number of samples classified correctly as no or failure.
3. False Positive (FP): Number of samples classified incorrectly as a yes or success.
4. False Negative (FN): Number of samples classified incorrectly as no or failure.

These values can be used to calculate the testing accuracy, sensitivity, and specificity as shown in the formulas below:

(3) $$Accuracy=\frac{TP+TN}{TP+TN+FP+FN},$$

(4) $$Sensitivity=\frac{TP}{TP+FN},$$

(5) $$Specificity=\frac{TN}{TN+FP}.$$

As shown in Table 3, Model 14 achieved the best performance on all three datasets, obtaining accuracy of 100% and 98% in training and validation datasets, respectively, after training for 46 epochs, and an accuracy of 95% on the test set. It stacks two convolution layers with 32 filters in each, followed by a batch norm layer and a dropout layer with a rate of 50%, a hidden fully connected layer with 32 neurons, another series of batch norm and dropout layers, and the output layer.

### 4.6. Generalization Capabilities

As shown in Table 1, the test set comprised 6 healthy samples and 16 unhealthy samples. The proposed model (Model 14) labeled the healthy group samples correctly, TP = 6; however, incorrectly labeled one out of the 16 unhealthy samples, TN = 15 (out of 16). Therefore, the prediction accuracy of this model is:

(6) $$Accuracy=\frac{6+15}{6+15+0+1}=0.9545\;(or\;95.45\%).$$

**Figure 4:**
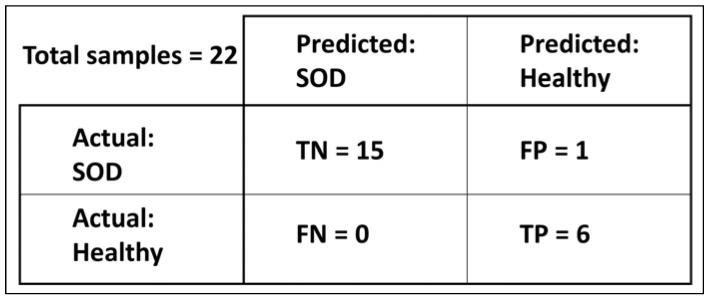
Confusion matrix summarizing the performance of the best-performing network on the test set. The matrix shows the number of true positives (TP), true negatives (TN), false positives (FP), and false negatives (FN) produced by the network on the test set.

The confusion matrix for the proposed network, Model 14, is shown in Figure 4. 

### 4.7. Ablation Study

The key components of the proposed network are convolution layers, batch normalization layers, dropout layers, and fully connected layers. An ablation study was conducted to verify the effects of each component on the model performance metrics [63,64]. Additionally, two types of adaptive optimizers, RMSProp (Root Mean Square Propagation) and Adam (Adaptive Moment Estimation) were investigated. Table 4 shows the performance metrics obtained by the different model architectures.

**Figure 5:**
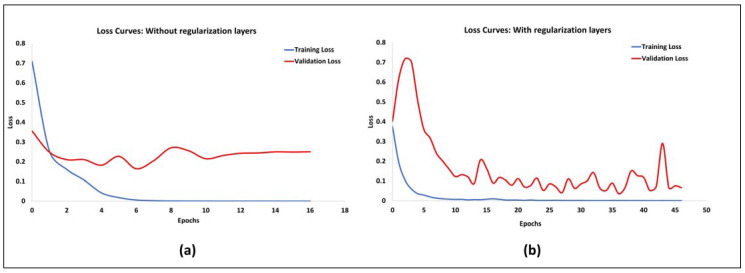
Comparison of the training and validation loss of the models with and without batch normalization and dropout layers. As observed in the left plot (**a**), the validation loss starts to increase with epochs while the training loss continues to decrease, demonstrating overfitting. This is addressed when the batch normalization and dropout layers (represented as regularization layers) are added, as demonstrated in the right plot (**b**).

Based on the results, the Adam optimizer (model A1) achieves a better performance as compared to the RMSProp optimizer (model A2). Comparing models A4–A7, adding a batch normalization layer and a dropout layer after the stacked convolution layers has a positive effect on the performance and improves overfitting. However, the overfitted models perform better on the test set. The loss curves obtained from training the networks with and without the batch normalization and dropout layers (models A1, and A7) are shown in Figure 5.

Model A4, where the batch normalization and dropout layers after the first fully connected layer are dropped, suggests that these layers have a negligible impact on the model’s performance on training and test sets. However, validation results are improved by adding these regularization layers.

This study validates that the originally proposed network (model A1) produces the best overall results with the given CVR dataset.

### 4.8. Transfer Learning Performance

The transfer learning experiments with EfficientNetB0, InceptionV3, and ResNet50 networks achieved training and validation accuracy in the range of 50–53%. With the early stopping criterion, these networks’ performance stopped improving after 27, 14, and 85 epochs, respectively. The VGG16 model produced the best results, achieving 99.83% and 94.88% training and validation accuracy, respectively, after training for 25 epochs.

All the pre-trained networks correctly labeled the unhealthy dataset. However, they incorrectly labeled the healthy dataset, classifying them as SOD class. Therefore, the overall prediction accuracy achieved by these networks is 72.72%.

### 4.9. Results Analysis

**Figure 6:**
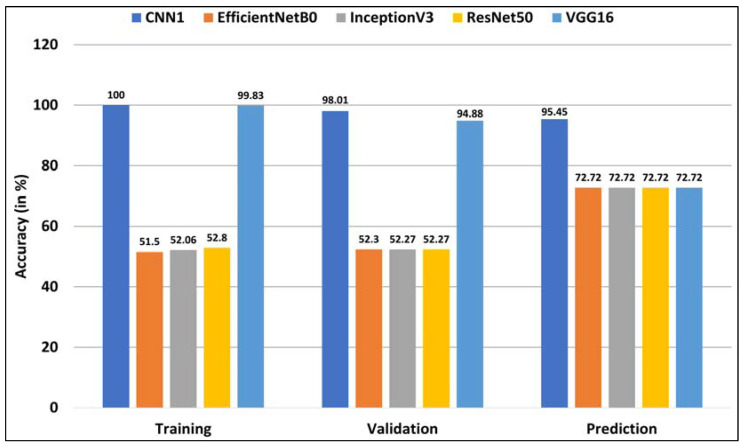
Comparison of the results obtained by the best-performing CNN trained from scratch (labeled as CNN1) and the pre-trained networks including EfficientNetB0, InceptionV3, ResNet50, and VGG16 on the training, validation, and test sets.

A comparison of the performance of the best-performing customized CNN trained from scratch and the transfer learning experiments is presented in Figure 6.

## 5. Discussion

### 5.1. Key Findings and Proposed Network

This study demonstrates that the convolution layers successfully identified the key parameters in the CVR maps, primarily the spatial distribution and intensity of reduced CVR, and the classifiers were able to discriminate between healthy control subjects and SOD patients. Based on the quantitative results obtained from the sensitivity analysis and the ablation experiments, it is evident that using a dropout layer after the convolution layer addressed overfitting and improved the model performance for the CVR dataset considered. Additionally, using a batch normalization layer further improved the overall model performance.

**Figure 7:**
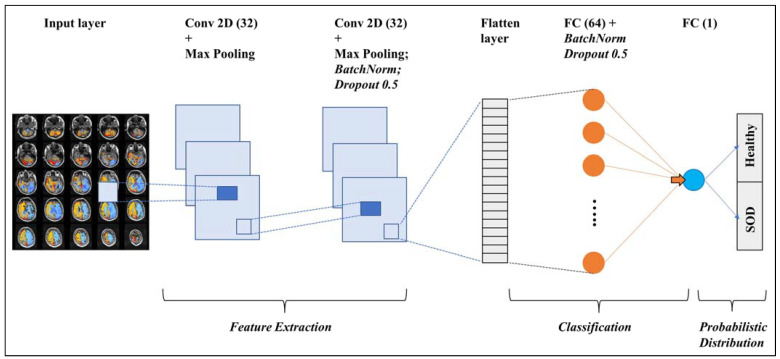
A schematic representation of the proposed CNN. The input layer is a matrix representation of the pixels in the input sample. Conv2D(32) represents a convolution layer with 32 filters, followed by a 2 × 2 2D max pooling layer. The second Conv2D layer has a batch normalization layer and a dropout layer after the max pooling layer. The features extracted by the convolution layers are fed into a flatten layer, which then goes into the fully connected layer with 64 nodes (represented as FC(64)). Finally, the output layer calculates a probabilistic distribution and classifies the input sample as a healthy subject or a SOD patient.

Based on the results obtained from the hyperparameter fine-tuning of customized CNNs that were trained from scratch, and using the pre-trained networks, a customized double-stacked CNN (Model 14 in Table 3) is proposed as the best-suited model for the CVR dataset used. The proposed network architecture is shown in Figure 7. The network architecture, weights, and biases were saved, and the model can be readily used to classify new CVR maps in the same mosaic configuration used in this study.

### 5.2. Limitations

**Figure 8:**
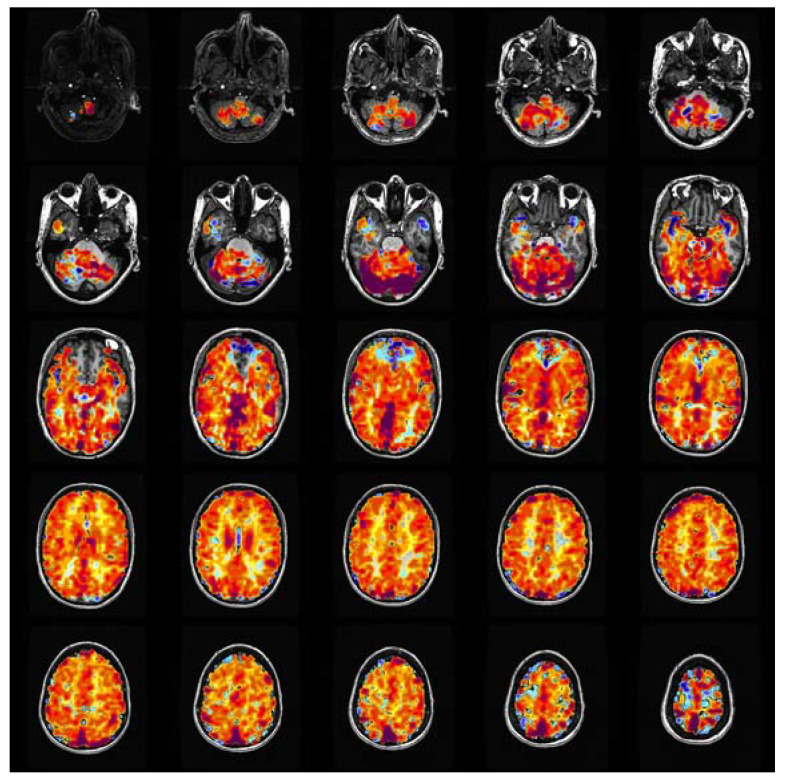
The input sample from the test set that was incorrectly labeled by the proposed CNN. As observed, there is no sign of steal physiology, indicated by the prominent red and yellow across the brain. The blue color is due to susceptibility artifacts.

The proposed CNN incorrectly labeled one sample (shown in Figure 8) in the test set, labeling a SOD patient as healthy. On review of the maps, while this patient has SOD, there is no steal physiology, indicating a normal CVR response. Therefore, although the CVR is “normal” for this patient, based on the objective of this study, this is an incorrect prediction. Such edge cases would require validation from experts.

Additionally, the proposed network is constrained by the color scale of the CVR maps, spacing between the brain slices while creating the mosaic images, and limited to 5 × 5 mosaic configurations. For other institutions to use this network, the same CVR analysis protocol would be needed. 

### 5.3. Future Work

As stated in Figure 1, the CVR maps used often have additional blue regions due to susceptibility artifacts that mimic steal. Since the key difference between healthy and unhealthy maps is the spatial distribution of reduced CVR, these artifacts may potentially influence the learning process. However, in the samples used in this study, these artifacts consistently appear in the anterior inferior frontal lobes from the paranasal sinuses, and in the temporal lobes from the mastoid air cells. This consistency allowed the CNNs to produce results with high accuracy. Future studies could investigate the impact of these artifacts on the model performance. Additionally, cross-validation should be implemented to validate the stability of the proposed network and its generalization capabilities.

For furthering this research, it is important to collect more data. The proposed network can be retrained with more samples to improve performance and extend generalization capabilities. In addition to SOD, there are other cerebrovascular diseases such as Alzheimer’s disease, Moyamoya disease, sickle cell disease, and small vessel disease, that can be highlighted using CVR studies [7,65]. For this multi-classification study, the data samples were insufficient to train the networks, but as clinical studies continue, CNNs can be implemented for screening these diseases. 

The transfer learning experiments, except for VGG16, yielded poor results with the CVR dataset used. The scarcity of training samples, the large number of parameters, and the complexity of the pre-trained networks could be the reasons. VGG16 is simpler and shallower as compared to others and therefore produced significantly better results. In fact, the proposed customized network uses a similar stacking of convolution layers as in the VGG architecture. Several studies have found immense success with transfer learning and there is scope to further the experimentations conducted in this research, including dropping more layers to reduce the learning parameters, changing the classifiers, and re-training them with additional samples as the dataset increases, to improve the results. Another implementation would be to employ the pre-trained networks as a feature extractor followed by a gradient-boosting tree classifier.

The scope of this study did not include investigating the generalization capabilities of the proposed network on a different set of CVR maps, obtained using a different vasoactive stimulus, for example, breath-hold, fixed inspired CO_2_, and chemical stimuli. As discussed earlier, research has shown that such transfer learning experiments with closely related datasets can achieve better performance than training a new network from scratch.

Finally, the use of CVR images in the Montreal Neurological Institute (MNI) coordinate space can be investigated, which would standardize the research, furthering the generalization capabilities of Artificial-Intelligence-driven CVR analyses.

## 6. Conclusions

The objective of this research was to further the current state of the application of deep learning in medical imaging by developing a clinical decision support system that can facilitate the screening of steno-occlusive disease (SOD) patients using cerebrovascular reactivity (CVR) maps. The CVR maps were obtained from clinical breath-control studies using prospective end-tidal targeting to administer a controlled P_ET_CO_2_ stimulus, accomplished using the RespirAct device. Image augmentation techniques were used to increase the number of input samples.

An empirical evaluation-based network optimization strategy was implemented for the customized convolutional neural networks (CNNs), and transfer learning was investigated by importing and modifying some of the popular pre-trained networks. Based on the experiments, a customized shallow CNN with two convolution layers and one hidden fully connected layer is proposed as the most optimal classifier. Results conclude that the proposed network successfully identifies the key features in the dataset considered to discriminate between healthy control subjects and patients with SOD. The generalization capabilities of this network are consistent with expert clinical readings, with only one incorrect prediction out of the 22 samples in the test set. 

While the proposed network is not production-ready, it can be used as a research tool to facilitate clinical decision-making and support CVR research advancements.

## Figures and Tables

**Table 1:** The total number of samples in the train, validation, and test sets after data augmentation was applied to the original input data.

Dataset	Healthy Samples	Unhealthy Samples
Train	594	650
Validation	168	186
Test	6	16

**Table 2:** The different hyperparameter settings that were experimented with as a part of the network architecture optimization process. The best values for each hyperparameter for the dataset considered are shown in bold font, wherever possible. Note: The hyperparameter combinations used were based on the results from sensitivity studies and therefore not all combinations are discussed.

Hyperparameter	Values
Batch Size	8, 16, **32**, 64
Batch Normalization	**Yes**/No (with default settings)
Dropout Rate	0% (No dropout), 20%, **50%**
Network Depth (number of layers)	2, 3 hidden layers
Network Width (filters/nodes in each layer)	8, 16, 32, 64, 128
Learning Rate	0.01, **0.001 (default)**, 0.0005
Kernel Size of Convolution Layer	**3 × 3**, 5 × 5, 7 × 7

**Table 3:** Results obtained on the training, validation, and test datasets by the different network architectures. In the table, convX represents a convolution layer with “X” filters, fcX represents a fully connected layer with “X” nodes, drop represents a dropout layer (rate = 0.5), and BN represents a batch normalization layer. Note: Each convolution layer is followed by a 2 × 2 2D max pooling layer. The best performing network is highlighted in bold font.

Model Number: Architecture	No. of Parameters	Epochs	Training Accuracy & Loss	Validation Accuracy & Loss	Testing Accuracy|Sensitivity & Specificity
1: conv8, fc8, drop, fc1	1,032,497	16	0.8977 & 0.21	0.8835 & 0.29	0.95|0.83 & 1
2: conv8, BN, drop, fc8, BN, drop, fc1	1,032,561	42	0.9670 & 0.06	0.97 & 0.09	0.72|1 & 0.62
3: conv16, BN, drop, fc16, BN, drop, fc1	4,129,633	14	0.9991 & 0.005	0.9204 & 0.177	0.77|0.17 & 1
4: conv16, BN, drop, fc32, BN, drop, fc1	8,258,561	53	0.8531 & 0.3465	0.9176 & 0.3149	0.27|1 & 0
5: conv16, BN, drop, fc64, BN, drop, fc1	16,516,673	16	1.00 & 0.0007	0.9631 & 0.1581	0.27|1 & 0
6: conv32, BN, drop, fc32, BN, drop, fc1	16,517,313	10	1.00 & 0.0054	0.5284 & 0.8745	0.27|1 & 0
7: conv32, BN, drop, fc64, BN, drop, fc1	33,033,601	10	1.00 & 0.0018	0.5250 & 2.3896	0.32|1 & 0.06
8: conv64, drop, fc64, drop, fc1	66,066,305	23	0.9867 & 0.0316	0.9488 & 0.2335	0.95|0.83 & 1
9: conv64, fc128, drop, fc1	132,131,585	12	1.00 & 0.0018	0.5284 & 1.1150	0.63|1 & 0.5
10: conv8, conv8, BN, drop, fc8, BN, drop, fc1	246,905	31	0.9884 & 0.0285	0.9204 & 0.2554	0.95|0.83 & 1
11: conv8, conv8, drop, fc16, drop, fc1	492,969	36	0.9975 & 0.0191	0.9318 & 0.19	0.31|1 & 0.06
12: conv16, conv16, BN, drop, fc16, BN, drop, fc1	986,993	40	1.00 & 0.0043	0.9715 & 0.0776	0.81|1 & 0.75
13: conv16, conv16, BN, drop, fc32, BN, drop, fc1	1,971,153	27	0.9983 & 0.0095	0.9176 & 0.3017	0.68|1 & 0.64
**14: conv32, conv32, BN, drop, fc32, BN, drop, fc1**	**3,946,721**	**46**	**1.00 & 0.0006**	**0.9801 & 0.0671**	**0.95|1 & 0.9375**
15: conv64, conv64, BN, drop, fc64, BN, drop, fc1	15,784,385	10	1.00 & 0.0024	0.7642 & 0.5937	0.7272|1 & 0.625

**Table 4:** Ablation study to verify the effect of each component of the proposed network on the overall performance. The model performance on the training, validation, and test sets are presented. In the table, convX represents a convolution layer with “X” filters, fcX represents a fully connected layer with “X” nodes, drop represents a dropout layer (rate = 0.5), and BN represents a batch normalization layer. Adam and RMSProp are the two optimizers investigated during the ablation study.

Model Name: Architecture	Epochs	Training Accuracy & Loss	Validation Accuracy & Loss	Testing Accuracy|Sensitivity & Specificity
A1: conv32, conv32, BN, drop, fc32, BN, drop, fc1; Adams	46	1.00 & 0.0006	0.9801 & 0.0671	0.95|1 & 0.9375
A2: conv32, conv32, BN, drop, fc32, BN, drop, fc1; RMSProp	19	0.9983 & 0.004	0.9090 & 0.5402	0.82|0.5 & 0.9375
A3: conv32, conv32, fc32, BN, drop, fc1	28	1.00 & 0.003	0.9232 & 0.26	0.95|1 & 0.9375
A4: conv32, conv32, BN, drop, fc32, fc1	64	1.00 & 0.075	0.9488 & 0.226	1|1 & 1
A5: conv32, conv32, BN, fc32, fc1	19	1 & 0.0004	0.9062 & 0.28	1|1|1
A6: conv32, conv32, drop, fc32, fc1	19	1 & 0.0006	0.929 & 0.15	0.95|1|0.9375
A7: conv32, conv32, fc32, fc1	16	1.00 & 0.00009	0.9233 & 0.25	1|1 & 1
A8: conv32, conv32, fc32, BN, fc1	19	1.00 & 0.0003	0.906 & 0.28	1|1|1
A9: conv32, conv32, fc32, drop, fc1	19	1.00 & 0.0006	0.93 & 0.15	0.95|1|0.9375

## Data Availability

The datasets and programs used and developed in this article are not readily available because of confidentiality agreements. Anonymized data and programs will be shared by request from any qualified investigator for purposes such as replicating procedures and results presented in the article provided that data transfer is in agreement with the University Health Network and Health Canada legislation on general data protection regulation. Requests to access the datasets should be directed to Olivia Sobczyk, olivia.sobczyk@uhn.ca.
